# Metabolic engineering of omega-3 long chain polyunsaturated fatty acids in plants using different ∆6- and ∆5-desaturases co-expressed with LPCAT from the marine diatom *Phaeodactylum tricornutum*

**DOI:** 10.1038/s41598-024-60141-3

**Published:** 2024-04-25

**Authors:** Sylwia Klińska-Bąchor, Kamil Demski, Yangmin Gong, Antoni Banaś

**Affiliations:** 1 Intercollegiate Faculty of Biotechnology, University of Gdańsk and Medical University of Gdańsk, ul Abrahama 58, 80-307 Gdańsk, Poland; 2 Department of Plant Breeding, Swedish University of Agricultural Sciences, Box 190, 23422 Lomma, Sweden; 3 Oil Crops Research Institute of Chinese Academy of Agricultural Sciences, Wuhan, 430062 China

**Keywords:** Plant sciences, Plant biotechnology, Fatty acids, Biochemistry, Lipids, Metabolic engineering

## Abstract

Continuous research on obtaining an even more efficient production of very long-chain polyunsaturated fatty acids (VLC-PUFAs) in plants remains one of the main challenges of scientists working on plant lipids. Since crops are not able to produce these fatty acids due to the lack of necessary enzymes, genes encoding them must be introduced exogenously from native organisms producing VLC-PUFAs. In this study we reported, in tobacco leaves, the characterization of three distinct ∆^6^-desaturases from diatom *Phaeodactylum tricornutum*, fungi *Rhizopus stolonifer* and microalge *Osterococcus tauri* and two different ∆^5^-desaturases from *P. tricornutum* and single-celled saprotrophic eukaryotes *Thraustochytrium sp*. The *in planta* agroinfiltration of essential ∆^6^-desaturases, ∆^6^-elongases and ∆^5^-desaturases allowed for successful introduction of eicosapentaenoic acid (20:5^∆5,8,11,14,17^) biosynthesis pathway. However, despite the desired, targeted production of ω3-fatty acids we detected the presence of ω6-fatty acids, indicating and confirming previous results that all tested desaturases are not specifically restricted to neither ω3- nor ω6-pathway. Nevertheless, the additional co-expression of acyl-CoA:lysophosphatidylcholine acyltransferase (LPCAT) from *Phaeodactylum tricornutum* boosted the proportion of ω3-fatty acids in newly synthesized fatty acid pools. For the most promising genes combinations the EPA content reached at maximum 1.4% of total lipid content and 4.5% of all fatty acids accumulated in the TAG pool. Our results for the first time describe the role of LPCAT enzyme and its effectiveness in alleviating a bottleneck called ‘substrate dichotomy’ for improving the transgenic production of VLC-PUFAs in plants.

## Introduction

Very-long-chain polyunsaturated fatty acids (VLC-PUFAs) are a group of fatty acids, which possess more than 20 carbons in length and three or more double bonds in the *cis* conformation^1^. In animal cells they maintain structural integrity, control cellular fluidity and contribute to cell signaling. Moreover, they constitute a very essential dietary source for mammals as their supplementation correlates with lower risk of developing several diseases, e.g. neurodegenerative or cardiovascular disorders^2^. Among VLC-PUFAs two groups of fatty acids are distinguished: ω3 and ω6, where omega number depends on the position of the first double bond from the methyl end of the carbon chain, in case of ω3 and ω6 the third and the sixth carbon, respectively. They vary in health benefits, as ω3-VLC PUFAs are known for their anti-inflammatory benefits, whereas predominance of ω6-VLC PUFAs in diet lowers nutritional value of consumed oils and may lead to adverse effects on health.

Major representants of ω6- and ω3-fatty acids, which are present in high quantity in plants are respectively linoleic (LA; 18:2^∆9,12^) and α-linolenic acid (ALA; 18:3^∆9,12,15^). Both fatty acids are major precursors of further VLC-PUFA biosynthesis in humans and animals. However, these organisms need to supplement LA and ALA exogenously, as their bodies do not possess neither ∆^12^- nor ∆^15^-desaturases. Nevertheless, mammals can conduct further conversion of VLC-PUFAs and produce eicosapentaenoic acid (EPA; 20:5^Δ5,8,11,14,17^) and docosahexaenoic acid (DHA; 22:6^Δ4,7,10,13,16,19^), however the efficiency of this process is very low and insufficient to cover full dietary demands^1,3^.

The most common source of EPA and DHA are marine fish, but this source is problematic because of several reasons. The most serious one is overfishing, even though, paradoxically, the current EPA and DHA acquirement from fish still does not meet the increasing global dietary requirements of the two fatty acids. Global warming is likely to cause the nutrient gap to increase^4,5^. Another issue might be potential environmental pollution of marine ecosystems^6^ and inefficient and unprofitable requirements of aquaculture supplementation in VLC-PUFAs, as fish are not native producers of these compounds^1^. Photosynthetic microalgae, a type of plankton is the primary link in the food chain and natural producers of VLC-PUFAs. However, large-scale production of microalgae is insufficient and prohibitive e.g. due to production costs and the need for large-scale cultivation technology. Therefore, their direct inclusion into an average human diet is unlikely.

**Figure 1:**
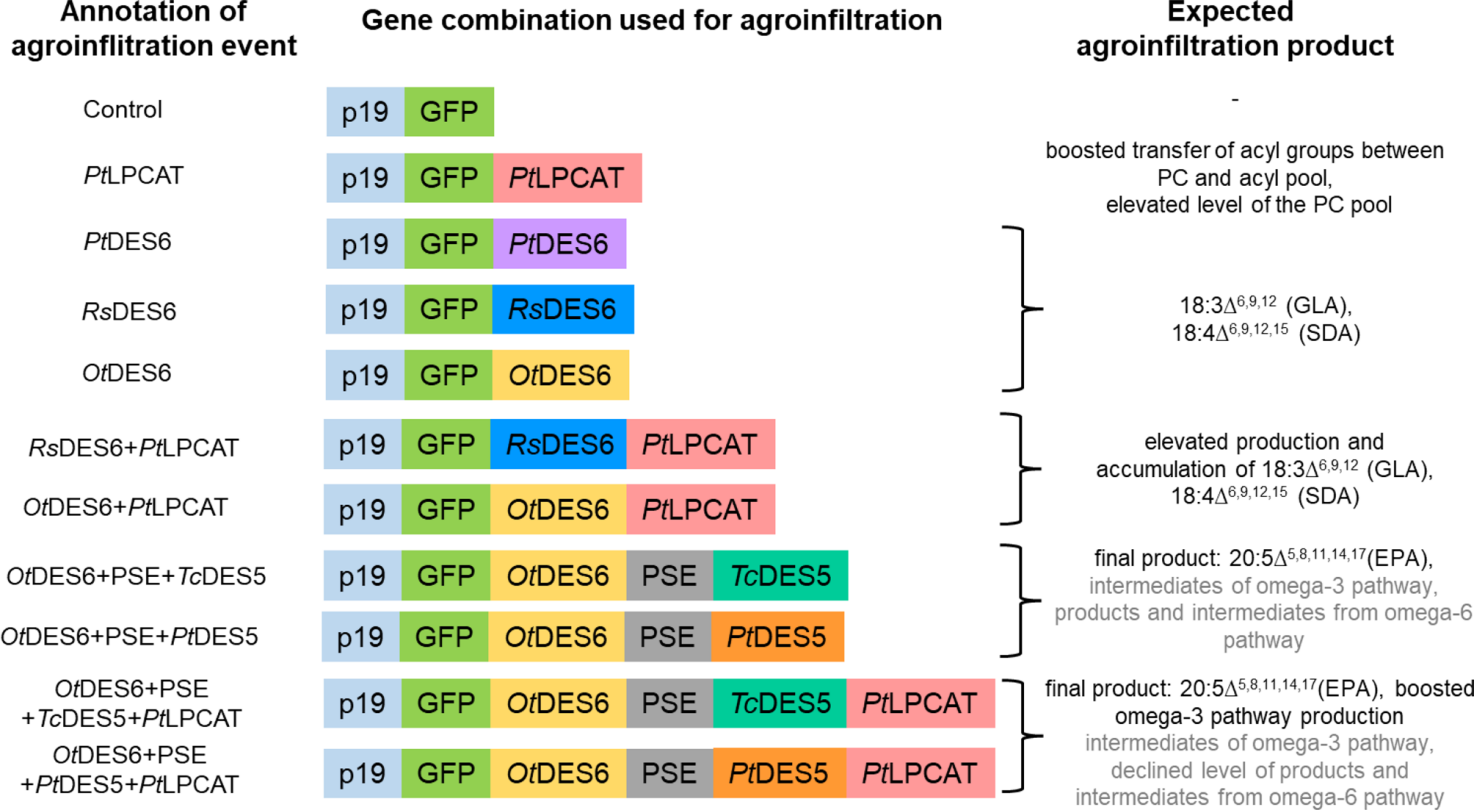
Outline of different gene combinations introduced in *Nicotiana benthamiana* transient expression experiments. *p19* silencing suppressor, *GFP* green fluorescent protein, *PtLPCAT* acyl-CoA:lysophosphatidylcholine acyltransferase from *Phaeodactylum tricornutum*, *PtDES6* ∆^6^-desaturase from *Phaeodactylum tricornutum*, *RsDES6* ∆^6^-desaturase from *Rhizopus stolonifer*, *OtDES6* ∆^6^-desaturase from *Osterococcus tauri, PSE* ∆^6^-elongases from *Physcomitrium patens*, *PtDES5* ∆^5^-desaturases from *Phaeodactylum tricornutum*, *TcDES5* ∆^5^-desaturases from *Thraustochytrium sp*.

**Figure 2:**
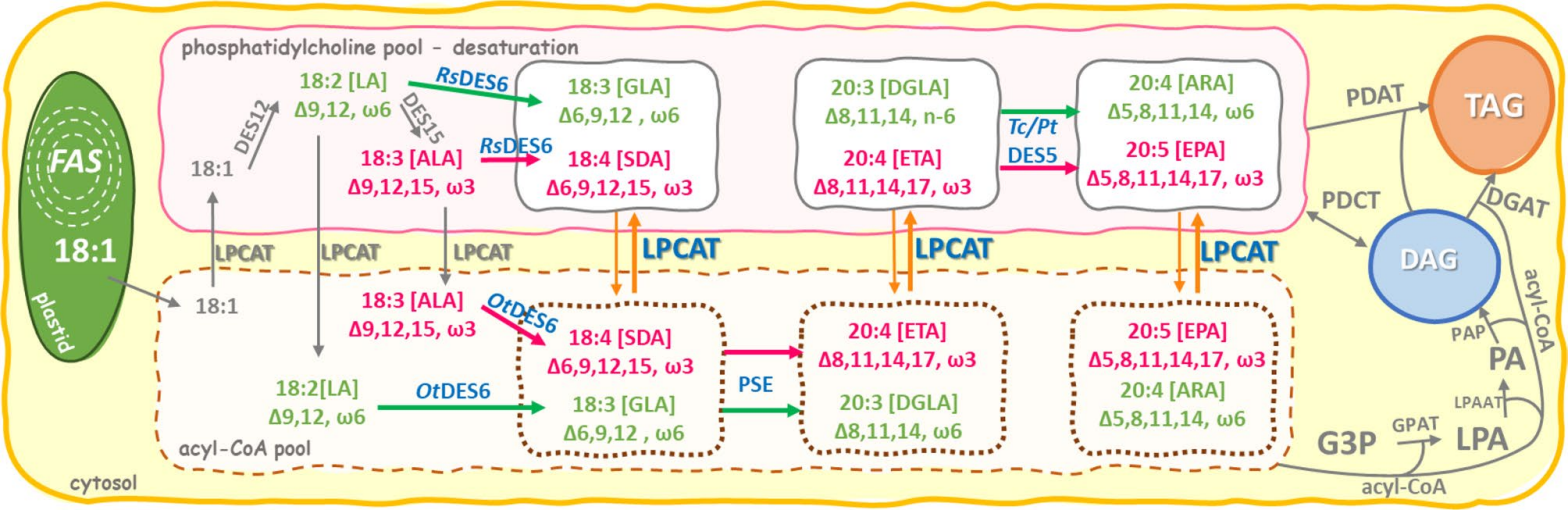
Overview of omega-3 and omega-6 VLC-PUFA biosynthesis pathway: products and intermediates of all designed combinations with genes used for Agrobacterium infiltration. Enzymes marked in blue represent the introduced enzymatic route. Grey arrows and names of enzymes denote endogenous enzymatic actions in *Nicotiana benthamiana.* Pink arrows represent omega-3 and green arrows indicate omega-6 biosynthesis pathway.

A possible alternative, which currently is being thoroughly investigated, is the incorporation of ω3 VLC-PUFA-producing enzymatic pathways into plants, with particular emphasis on oilseed plants. As mentioned previously, plants are the main reservoir of LA and ALA, but they lack specific desaturases and elongases involved in the conversion of both fatty acids into VLC-PUFAs. Biosynthesis of these fatty acids via classical ω 6- and ω3-pathways consists of successive reactions catalyzed by ∆^6^-desaturases, ∆^6^-elongases and ∆^5^-desaturases leading to EPA production (see Figs. 1 and 2). The ∆^6^-desaturase is responsible for the synthesis of 18:3^∆6,9,12^ (GLA; γ-linolenic acid; ω6) or 18:4^∆6,9,12,15^ (SDA; stearidonic acid; ω3) from LA or ALA, respectively. Further, reaction of elongation extends the carbon chain by two carbons and leads to the production of: 20:3∆^8,11,14^ (DGLA; dihomo-γ-linolenic acid; ω6) and 20:4^∆8,11,14,17^ (ETA; eicosatetraenoic acid; ω3). Second route of desaturation via ∆^5^-desaturase results in 20:4^∆5,8,11,14^ (ARA; arachidonic acid; ω6) and EPA synthesis from DGLA and ETA, respectively. Further production of DHA is catalyzed by ∆^5^-elongase and ∆^4^-desaturase. Additionally, each of the fatty acids synthesized via omega-6 pathway can be converted to omega-3 fatty acids via an appropriate, innate ω3 desaturase.

Until now, many attempts have been made to introduce the VLC-PUFAs biosynthesis pathway in plants by incorporating gene encoding essential enzymes natively presented in producer organisms. The most promising results were obtained in *Camelina sativa* seeds, which composition was comparable to VLC-PUFAs in fish oil^7^. Nevertheless, even more effective combinations of enzymes leading to VLC-PUFAs production are being sought after. One issue that has remained unsolved for many years is a bottleneck concerning the problem called ‘substrate dichotomy’^8,9^. It appears as a consequence of diverse substrate requirements for desaturases and elongases. The overwhelming majority of desaturases utilize phospholipid-linked fatty acids whereas elongases use acyl-CoA as substrate. The postulated option to overcome this obstacle is the co-expression of a designed VLC-PUFAs biosynthesis cassette with acyl-CoA:lysophosphatidylcholine acyltransferase (LPCAT);^1,8,9^. This might alleviate the bottleneck as this enzyme is responsible for bidirectional transfer of acyl group from phosphatidylcholine to cytosolic pool of acyl-CoA^10^, making necessary intermediates for VLC-PUFAs more accessible for desaturase and elongases. The studies conducted so far indicate that natively present in Arabidopsis and flax seed LPCAT activity with non-native Δ^6^-polyunsaturated fatty acids is limited^8,11^, therefore expression of LPCAT from an organism that naturally produces VLC-PUFAs seems promising.

To answer this question, we conducted experiments based on simultaneous co-expression with gene combinations aimed for EPA production and LPCAT from *Phaeodactylum tricornutum*, which led us to successful *in planta* incorporation of EPA biosynthesis pathway. Previously, we characterized substrate specificities of the used LPCAT, which has been found to esterify 18:3, 18:4 and 20:4 (ω3) acyl groups from acyl-CoA to lysophosphatidylcholine. To our knowledge it is the only LPCAT characterized from marine microorganisms^12^, and it’s a first attempt to introduce it into plant organisms. Additionally, during this research we determined activities of three ∆^6^-desaturases from *Osterococcus tauri* (specific to acyl-CoAs)^7,11,13,14^, *Rhizopus stolonifer*^15^, *P. tricornutum*^11,16^; (the latter two ones both phospholipid-linked desaturases) and, ∆^6^-elongases from *Physcomitrium patens*^17^ and two ∆^5^-desaturases from *P. tricornutum*^11,16^ and *Thraustochytrium sp*.^18^, which were studied in gene combinations with or without co-expression with *Pt*LPCAT^12^.

## Results

### Genetic design for omega-3 fatty acid production *in planta*

The aim of our research was to investigate the incorporation of the VLC-PUFA biosynthesis pathway with special focus on efficient production of EPA. These compounds and their intermediates are foreign to most plant organisms. Achieving such a goal requires substantial modification of the biosynthesis pathway of fatty acids, which is made possible by introducing different combinations of specific genes necessary to introduce new enzymatic activities of this pathway (Fig. 1).

The first task was to verify activity of different ∆^6^-desaturases and achieve efficient production of 18:3∆^6,9,12^ (GLA) and/or mostly 18:4∆^6,9,12,15^ (SDA), as a result of the first step of omega-3 VLC-PUFAs production. The *Rs*DES6 and *Pt*DES6 desaturases from filamentous fungus *R.stolonifer* and diatom *P. tricornutum*, respectively, are phospholipid-linked desaturases, therefore they can utilize fatty acids that have been previously transferred into phosphatidylcholine. On the other hand, the *Ot*DES6 desaturase from the green microalga *O. tauri* is an acyl-CoA Δ^6^-desaturase. It utilizes fatty acids directly from the cytosol pool, and its reaction products are more accessible for elongases than those of the other two studied desaturases (Fig. 2).

Since the transfer of PUFAs between PC pool and the cytoplasmic pool of acyl-CoA is thought to be a potential bottleneck in the efficient omega-3 production in plants, we decided to verify this hypothesis by implementing expression of LPCAT from *P. tricornutum* (*Pt*LPCAT), which is responsible for transferring an acyl group from cytosol to the PC pool, and vice-versa (Fig. 2). The gene was used separately and in combinations with ∆^6^-desaturases to check, if additional expression of *Pt*LPCAT can boost production and accumulation of GLA and SDA.

The final task was to introduce the whole omega-3 VLC-PUFA biosynthesis pathway. The most promising ∆^6^-desaturase (*Ot*DES6) was combined with ∆^6^-elongase from moss *P. patens* and two different ∆^5^-desaturases: *Tc*DES5 from single-celled saprotrophic eukaryote *Thraustochytrium sp.* And *Pt*DES5 from *P. tricornutum*, both being lipid-linked desaturases.

The desired, final product of these gene combinations is EPA, but due to substrate dichotomy and anticipated partial incompatibility of the introduced enzymatic activities with the plant system, we expected that both the ARA pathway products and the EPA pathway intermediates would accumulate to a significant extent. To boost the production of EPA and minimize production of omega-6 compounds, we combined the mentioned multigene combinations with *Pt*LPCAT. This enzyme shows higher substrate preference toward omega-3 than toward omega-6 intermediates. The plant LPCAT has not shown such specificity^12^.

To produce desired compounds from the described combinations, we codon-optimized each gene sequence for plant expression. Final gene expression cassettes were hosted by separate Agrobacterium, which were used for *Nicotiana benthamiana* leaves infiltration. As a control infiltration, leaf infected with Agrobacterium caring green fluorescent protein (GFP) was used as a reporter gene and the p19 silencing suppressor has been used as well.

### Screening of fungal and algal ∆^6^-desaturase activity and diatom LPCAT activity for ω3 fatty acid synthesis in ***Nicotiana benthamiana***

**Figure 3:**
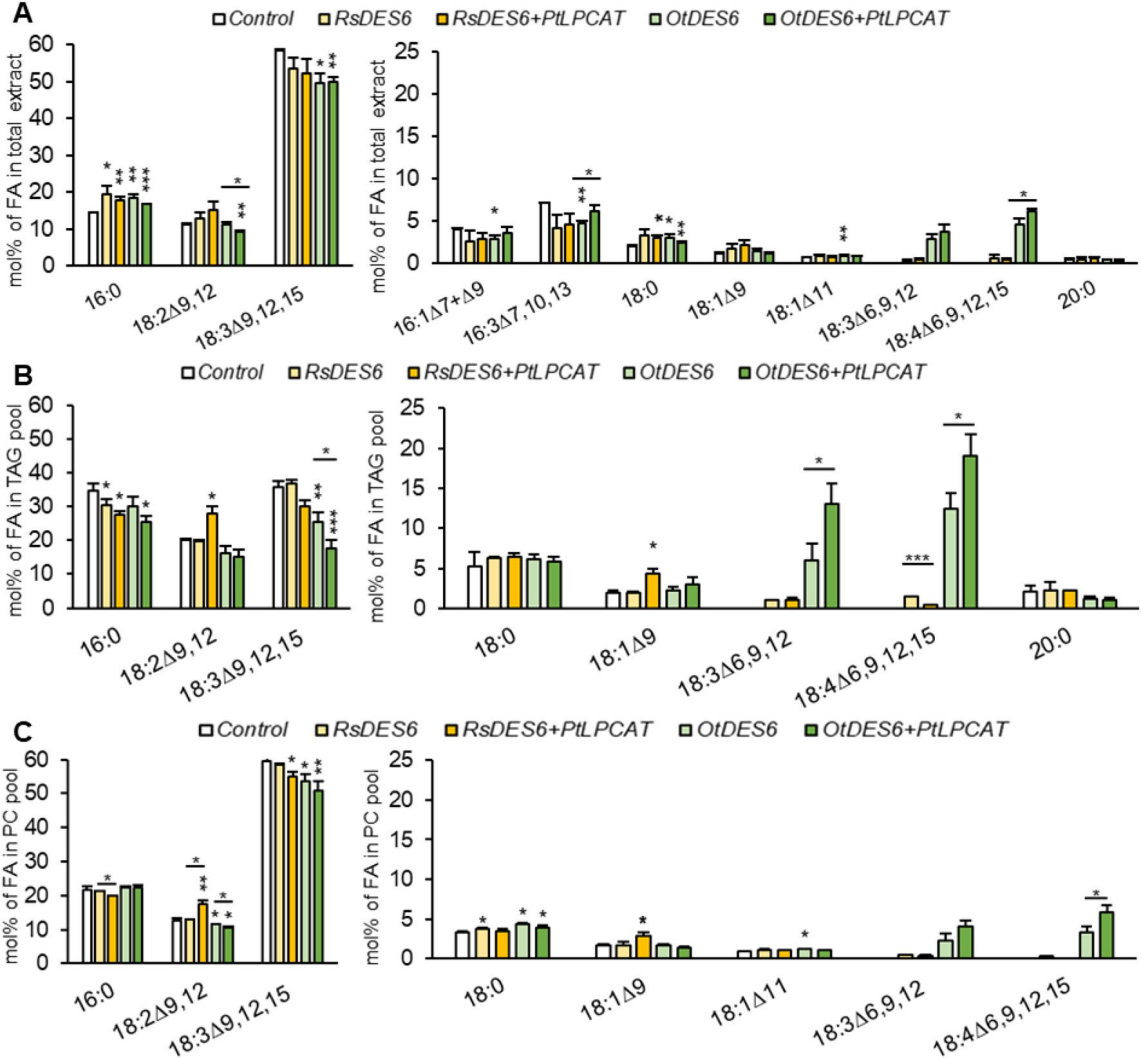
Fatty acid composition in acyl-lipid (**A**), triacylglycerol (**B**) and phosphatidylcholine pools (**C**) of *Nicotiana benthamiana* leaves obtained through agroinfiltration with gene combinations aimed at producing GLA (18:3∆^6,9,12^) and SDA (18:4∆^6,9,12,15^). The result concern the effect of *Rs*DES6, *Ot*DES6 action and the outcome of their co-expression with *Pt*LPCAT. Error bars present standard deviations between independent biological replicates. Asterisk above bars denote statistical significance compared to control and asterisks above the line indicate statistical difference between combination with desaturase and desaturase with *Pt*LPCAT. Statistical significance was calculated in two-tailed Student’s t-test: *p ≤ 0.05; **p ≤ 0.01; ***p ≤ 0.001.

To search for the most promising ∆^6^-desaturase, we first tested three enzymes from different organisms. Only two of the three turned out to be active and exhibited the ability to produce substantial amounts of ALA and SDA in *N. benthamiana* leaves (Fig. 3a). For further analysis we used desaturase from *O. tauri*, which activity and substrate preference were previously confirmed in both seeds and yeast^7,11,13,14^, and *R. stolonifer*, which was only characterized in yeast^15^. Incorporation of *Ot*DES6 resulted in a sevenfold increase of GLA and SDA production compared to the effect of *Rs*DES6 action. Correspondingly, the content of GLA and SDA in total lipid extract amounted to 0.4% and 0.7% for *Rs*DES6 and 2.8% and 4.7% for *Ot*DES6 activity.

The third tested desaturases—*Pt*DES6 did not affect neither total lipid nor PC and TAG pool compositions (Table S1). In our preliminary assays in which the genetic construct contained nos promoter and *Pt*DES6 sequence and was not plant codon-optimized, we also did not notice any effect. Although the activity of this enzyme was confirmed earlier in yeast^11,16^, our data indicates that it is inactive in the vegetative tissue of tobacco.

**Figure 4:**
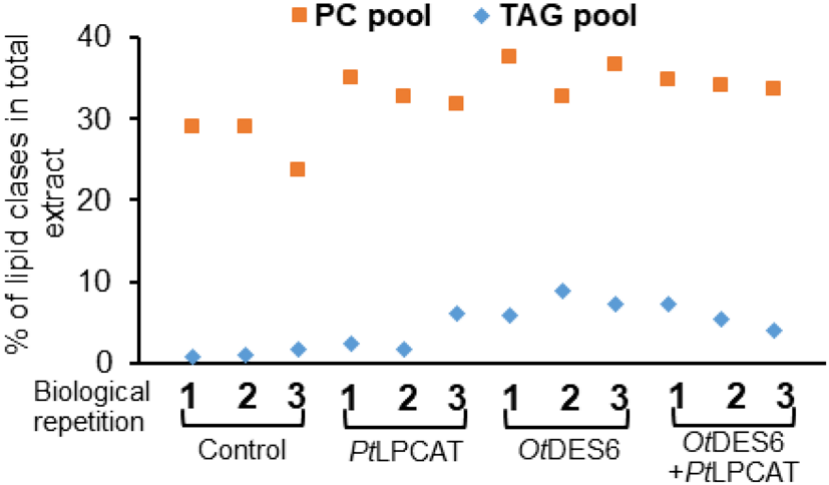
Distribution of phosphatidylcholine and triacylglycerol content in *Nicotiana benthamiana* leaves, caused by the action of three different exogenous gene combinations: *Pt*LPCAT, *Ot*DES6 and *Ot*DES6 with *Pt*LPCAT. The dots denote the content of the PC or TAG pools of each infiltrated sample (three for each gene combination), measured as the percentage of whole extract separated on TLC plates. The mean value and standard deviation are presented in Table S2.

*Pt*LPCAT action has been tested for the first time in plant tissue. Its expression did not significantly affect the composition of total lipid and PC pool, however it modified the amount of 18:3∆^9,12,15^ in the TAG pool (it increased from 36% in control to 43%; Table S1). Moreover, the PC pool, compared to control, increased by 5.9% and the TAG pool by 2.2% (from 27.3% to 33.2% and from 1.2 to 3.4%, respectively) (Fig. 4, Table S2).

### The effects of ∆^6^-desaturase from ***Rhizopus stolonifer*** expression and its co-expression with ***Pt***LPCAT

*R. stolonifer* is an endophytic fungus with a confirmed ability to produce GLA^15^. Our infiltrations of *N. benthamiana* leaves with p19, GFP and *Rs*DES6, indicated that *Rs*DES6 action leads to the production of both GLA and SDA, with more SDA than GLA being produced. Nevertheless, they both constituted a negligible pool of fatty acids. In the TAG pool they accounted for only 1% and 1.5% and in the PC pool 0.4% and 0.3%, respectively, when *Rs*DES6 were expressed separately. The addition of *Pt*LPCAT to this gene combination did not affect GLA accumulation, but strongly disturbed the content of SDA, which fell below the level of detection (in the PC pool); (Fig. 3bc).

Since, the synthesis of SDA is at a low level, any noteworthy difference in the accumulation of ALA, a substrate for SDA synthesis, was determined via *Rs*DES6 action. Only in combination with *Pt*LPCAT the amount of ALA declined by about 4% in the PC pool, about 6% in the TAG pool, and about 1.5% in total lipid content, although there was no significant increase in SDA content (Fig. 3). This change was also visible in the remaining lipid pool with exception of PC and TAG analyses (Table S3).

Primary substrate for GLA synthesis is LA. Despite the expected decrease in the content of LA we observed its elevated accumulation, when *Pt*LPCAT was co-expressed. In these gene combinations LA levels have significantly increased in the TAG pool, from 20.3 to 27.9%, and in the PC pool, from 12.7 to 17.6% (Fig. 3bc).

In total lipid content we noticed elevated content of saturated fatty acids: 16:0 and 18:0. For *Rs*DES6 and *Rs*DES6 + *Pt*LPCAT combinations, compared to control, their content rose by 4.9% and 1.7%, respectively. Elevated level of 18:0 was also detected in the PC pool, however the level of 16:0 remained comparable to control. Whereas, in the TAG pool, an inverse pattern for 16:0 was detected and reductions by 4.5% and 7.2% were counted, between control and *Rs*DES6 and control and *Rs*DES6 + *Pt*LPCAT, respectively (Fig. 3). The increasing palmitic acid content was also noticeable in the lipid fractions without the PC and TAG pools (Table S3), which indicates that this fatty acid needs to accumulate in other lipid classes e.g., galactolipids.

Analysis of fatty acid content in total extract allowed for detection of three fatty acids which are characteristic for plastid lipids: 16:1^∆7^, 16:1^∆9^ and 16:3^∆7,10,13^, which exhibited slightly reduced accumulation, however without statistical significance (Fig. 3a).

### The effects of ∆^6^-desaturase from ***Osterococcus tauri*** expression and its co-expression with ***Pt***LPCAT

*O. tauri*, the second organism, which ∆^6^-desaturase was characterized in our study, is a marine photosynthetic picoeukaryote being able to efficiently synthesize both eicosapentaenoic acid (20:5^Δ5,8,11,14,17^) and docosahexaenoic acid (22:6^Δ4,7,10,13,16,19^);^19^. Our analysis confirmed its activity; tobacco leaves agroinfiltrated with p19, GFP and *Ot*DES6 accumulated high levels of GLA and SDA, which accounted for 5.9% and 12.5% in TAG pool and 2.2% and 3.2% in PC pool, respectively (Fig. 3b,c). Co-expression with *Pt*LPCAT notably boosted the production of these fatty acids. In total lipid extract, the content of both fatty acids increased from 2.8 to 3.7% and from 4.7 to 6.1%, respectively for GLA and SDA content. In the PC pool their content increased 1.8 times for gene combinations with the aforementioned desaturase and *Pt*LPCAT co-expression. Whereas the TAG pool contained 1.8 times and 1.5 times more GLA and SDA, 13.1% and 19.1%, respectively. The obtained results, supported by statistical significance, strongly indicate a positive effect of *Pt*LPCAT action on production and accumulation of both fatty acids (Fig. 3a).

Elevated biosynthesis of GLA and SDA in the tested combinations resulted in a substantial reduction of LA and ALA. In total lipid extract content of LA declined (by about 20% of content detected for control) only in tobacco leaves infiltrated with the *Ot*DES6 + *Pt*LPCAT combination, compared to control (Fig. 3a). Similar results, but for both the *Ot*DES6 and the *Ot*DES6 + *Pt*LPCAT combinations were noticed in the TAG pools. In the PC pool, compared to control, LA decreased by 1.2% for *Ot*DES6, whereas in co-expression with *Pt*LPCAT LA decreased by 2.3%. ALA content diminished especially in the TAG pool (where the highest GLA and SDA accumulation were noticed). Agroinfiltration with only *Ot*DES6 reduced ALA levels by 10.2%, while combination of *Ot*DES6 with *Pt*LPCAT reduced levels of ALA by half of the amount in control tobacco leaves. For both ω3 fatty acid-directed combinations, total lipid extract and PC pool contained approximately 85–90% of ALA detected in control (Fig. 3). Decrease in the content of these fatty acids was also noticeable in remaining tested lipid pools (Table S3).

Accordingly, with the combination with desaturase from *R. stolonifer*, elevated accumulation of saturated fatty acids was detected (Fig. 3a). Similar patterns were noticed for 16:0 and 18:0 present in PC and TAG pool (Fig. 3b,c). In parallel, reduced content of plastid fatty acids in the total lipid content was observed, however without statistical significance.

Since both applied gene combinations resulted in efficient GLA and SDA production, we checked their effect not only on fatty acids composition but also determined the changes in overall content of the two tested lipid classes. Both combinations: *Ot*DES6 separately and co-expressed with *Pt*LPCAT boost the accumulation of PC and TAG pools. PC pool raised up from 27.3% detected for control to 35.7% and 34.2% for *Ot*DES6 and *Ot*DES6 + *Pt*LPCAT, respectively). The TAG pool content increased 4.6 times for *Ot*DES6 + *Pt*LPCAT and six times for *Ot*DES6, compared to control (Fig. 4, Table S2).

### EPA biosynthesis and accumulation through co-expression of ∆^6^-desaturase, ∆^6^-elongase, and two distinct ∆^5^-desaturases along with ***Pt***LPCAT

**Figure 5:**
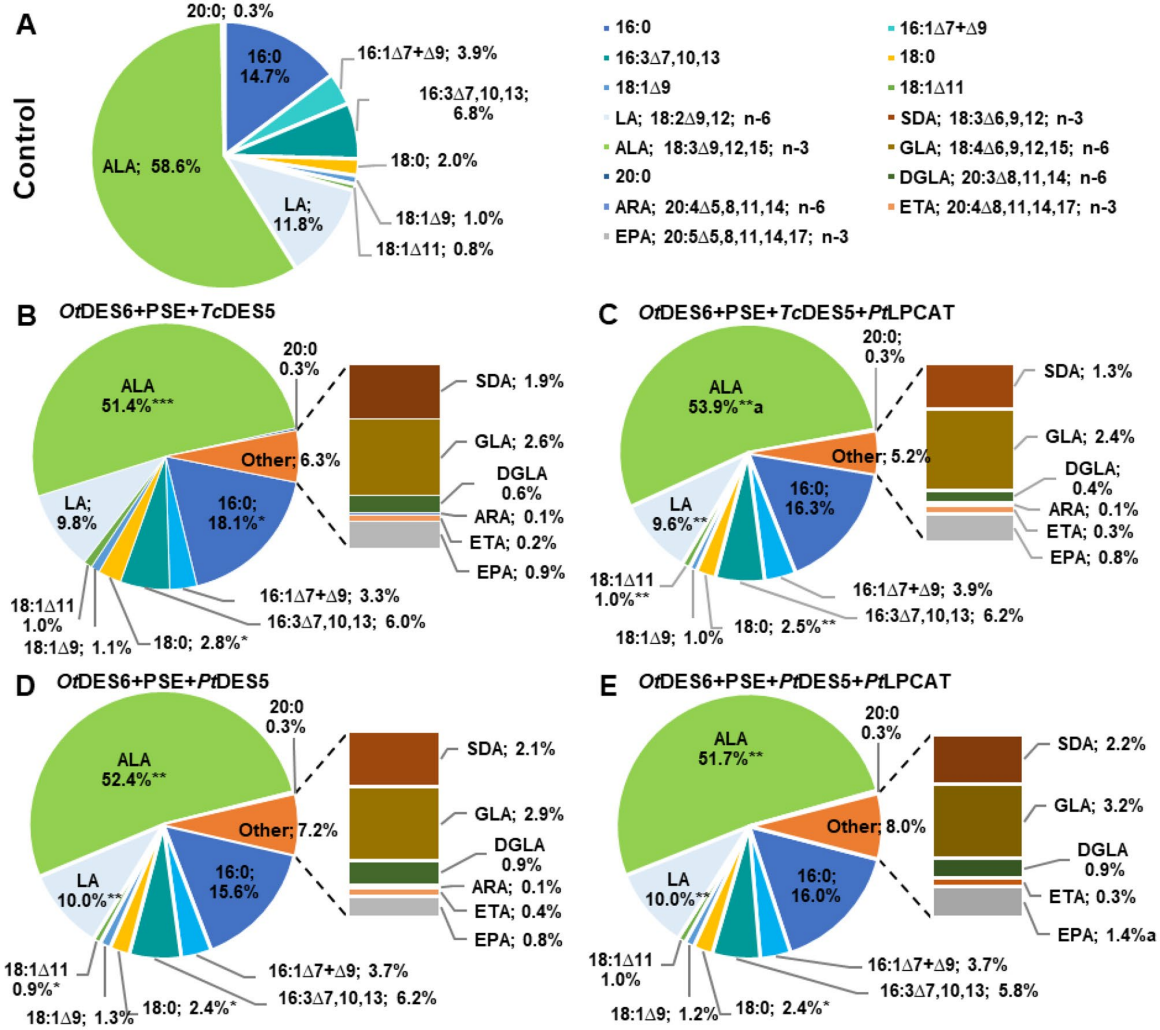
Fatty acid composition of acyl-lipids of *Nicotiana benthamiana* leaves obtained through agroinfiltration with gene combinations aimed at producing EPA (20:5^∆5,8,11,14,17^). Panel A represents the result for control (expression of p19 and GFP only). Panel B and C concern *Ot*DES6 + PSE + *Tc*DES5 and *Ot*DES6 + PSE + *Tc*DES5 + *Pt*LPCAT, respectively, whereas panels D and E represent *Ot*DES6 + PSE + *Pt*DES5 and their co-expression with *Pt*LPCAT, respectively. The values denoted at the pie charts are means of three independent biological replicates. Asterisks indicate statistical significance compared to control and letters indicate statistical difference between combination with and without *Pt*LPCAT. Statistical significance was calculated in a two-tailed Student’s t-test: *p ≤ 0.05; **p ≤ 0.01; ***p ≤ 0.001; ^a^p ≤ 0.05. Standard deviations are presented in Table S6.

Based on previous analysis we chose to further study ∆^6^-desaturase from *O. tauri*, as the one, which action led to high content of GLA and SDA, with higher SDA production (omega-3 fatty acid). Co-expression of this desaturase with PSE, *Pt*DES5 or *Tc*DES5 resulted in the production of EPA and other intermediates from both ω3 and ω6 pathways. Beside GLA, we detected DGLA and ARA belonging to ω6 fatty acids. However, DGLA was absent from the TAG pool for each tested combination. Similarly, ARA was missing from the total lipid content for one combination: *Ot*DES6 + PSE + *Pt*DES5 + *Pt*LPCAT. Fatty acids belonging to omega-3 fatty acids: SDA, ETA and EPA were identified for all combinations and in all tested lipid pools (Fig. 5; Table 1; Table S4).

**Table 1:** Fatty acid composition (mol%) in phosphatidylcholine and triacylglycerol pool of *Nicotiana benthamiana* leaves derived through agroinfiltration with gene combinations aimed at producing EPA (20:5^∆5,8,11,14,17^).

	Phosphatidylcholine pool											
	16:0	18:0	18:1^∆9^	18:1^∆11^	18:2^∆9,12^	18:3^∆6,9,12^	18:3^∆9,12,15^	18:4^∆6,9,12,15^	20:3^∆8,11,14^	20:4^∆5,8,11,14^	20:4^∆8,11,14,17^	20:5^∆5,8,11,14,17^
Control	18.8 ± 1.6	2.4 ± 0.6	1.0 ± 0.3	0.8 ± 0.2	8.6 ± 4.0	–	66.5 ± 7.0	–	–	–	–	–
*Ot*D6 + PSE + *Tc*D5	22.4 ± 0.4*	3.8 ± 0.1*	1.6 ± 0.3	1.2 ± 0.1*	11.6 ± 1.1	1.5 ± 0.7	51.9 ± 1.8*	2.0 ± 0.9	0.4 ± 0.2	0.2 ± 0.02	0.2 ± 0.1	0.5 ± -.3
*Ot*D6 + PSE + *Tc*D5 + *Pt*LPCAT	24.3 ± 2.2*	4.0 ± 0.2*	1.3 ± 0.3	1.2 ± 0.04	10.9 ± 1.6	1.1 ± 0.2	52.1 ± 1.1	1.8 ± 0.4	0.2 ± 0.07	0.1 ± 0.03	0.2 ± 0.1	0.3 ± 0.09
*Ot*D6 + PSE + *Pt*D5	21.7 ± 0.5	3.5 ± 0.2	1.7 ± 0.1*	1.2 ± 0.08*	12.0 ± 0.2	1.6 ± 0.7	50.7 ± 1.9	2.1 ± 0.7	0.9 ± 0.06	0.1 ± 0.03	0.3 ± 0.1	1.1 ± 0.2
*Ot*D6 + PSE + *Pt*D5 + *Pt*LPCAT	22.0 ± 0.5	3.5 ± 0.2*	1.6 ± 0.1*	1.2 ± 0.1*	11.7 ± 0.7	1.6 ± 0.7	51.2 ± 1.5	2.2 ± 0.8	0.8 ± 0.02	0.1 ± 0.02	0.2 ± 0.1	1.2 ± 0.1

	Triacylglycerol pool											
	16:0	18:0	18:1^∆9^	18:2^∆9,12^	18:3^∆6,9,12^	18:3^∆9,12,15^	18:4^∆6,9,12,15^	20:0	20:4^∆5,8,11,14^	20:4^∆8,11,14,17^	20:5^∆5,8,11,14,17^	
Control	37.6 ± 0.7	6.5 ± 0.5	2.0 ± 0.5	19.8 ± 0.5	–	32.6 ± 1.0	–	1.5 ± 0.4	–	–	–	
*Ot*D6 + PSE + *Tc*D5	34.8 ± 2.1	7.1 ± 0.9	1.7 ± 0.3	13.9 ± 1.1**	5.3 ± 0.7	18.6 ± 1.7**	5.9 ± 1.3	1.5 ± 0.3	4.0 ± 0.5	1.8 ± 0.4	5.5 ± 1.1	
*Ot*D6 + PSE + *Tc*D5 + *Pt*LPCAT	35.9 ± 1.4	6.5 ± 0.3	1.8 ± 0.8	14.3 ± 0.7**	3.2 ± 0.6_a_	22.9 ± 0.6**_a_	7.3 ± 0.5	1.6 ± 0.3	2.5 ± 1.1	1.0 ± 0.1	2.9 ± 0.9	
*Ot*D6 + PSE + *Pt*D5	27.4 ± 4.9	7.9 ± 1.0	2.5 ± 0.6	15.4 ± 1.5*	5.2 ± 1.1	20.0 ± 2.1*	8.5 ± 1.2	1.8 ± 0.1	2.7 ± 1.1	4.2 ± 0.5	4.3 ± 1.3	
*Ot*D6 + PSE + *Pt*D5 + *Pt*LPCAT	31.4 ± 1.8**	6.7 ± 0.2	2.0 ± 0.1	15.0 ± 1.1*	4.3 ± 1.7	21.6 ± 5.2**	7.6 ± 2.2	1.7 ± 0.1	2.1 ± 1.2	3.1 ± 1.2	4.5 ± 0.9	

Mean values and standard deviations of three independent biological replicates are presented. Asterisks denote statistical significance compared to control calculated in two-tailed Student’s t-test: *p ≤ 0.05; **p ≤ 0.01; ***p ≤ 0.001.

Although leaves are not a major reservoir of very-long chain fatty acids, the introduction of tested gene combinations led to production of omega-3 and omega-6 fatty acids at between 5.2 and 8.0% on average of all fatty acids present in total lipid extract. The combination of *Ot*DES6 + PSE + *Pt*DES5 + *Pt*LPCAT turned out to be the most effective. For this combination 49% of ‘Other’ fatty acids consisted of newly synthetized omega-3 fatty acids. The desired, final product—EPA accounted for 17.5% of the ‘Other’ fatty acids pool and in total lipid extract it accounted for 1.4% of all fatty acids (Fig. 5).

In the PC pool overall content of newly synthesized omega-3 fatty acids fluctuated between 3.7 and 6.1% of all fatty acids. Within all newly synthesized fatty acids, omega-3 fatty acids consist of 56% and 57% for *Ot*DES6 + PSE + *Tc*DES5 and *Ot*DES6 + PSE + *Pt*DES5 combinations, respectively. Whereas co-expression with *Pt*LPCAT increased their accumulation to 62% and 59% of all newly synthesized fatty acids, respectively (Table 1).

A similar pattern of higher accumulation of omega-3 fatty acids in newly synthesized fatty acids pool was noticed for TAG pool, where parallel co-expression with *Pt*LPCAT boosted their content by 7% (*Ot*DES6 + PSE + *Tc*DES5; from 59 to 66%) and by 2% (*Ot*DES6 + PSE + *Pt*DES5; from 68 to 70%). Despite that, the additional action of *Pt*LPCAT resulted in reduction of the newly synthesized fatty acid pool content by 25% for *Ot*DES6 + PSE + *Tc*DES5 + *Pt*LPCAT and by 13% for *Ot*DES6 + PSE + *Pt*DES5 + *Pt*LPCAT, compared to the combination without *Pt*LPCAT. Nevertheless, the content of EPA in the newly synthesized fatty acids pool reached 17% and 20.1% for this pool, (2.9% and 4.5% of all fatty acids in the TAG pool), for corresponding combinations (Table 1).

The distribution of the remaining detected fatty acids was like those observed when only ∆^6^-desaturases actions were tested. Since ALA and LA are primary precursors in omega-6 and omega-3 pathways, their content declined significantly in all tested lipid pools compared to the control combination (Fig. 5 and Table 1). On average ALA declined from 58.6% (control) by 6.3% and LA from 11.8% (control) by 2% in total lipid content, also affecting composition of PC and TAG. Again, the total lipid content was characterized by elevated accumulation of long-chain saturated fatty acids (16:0 and 18:0). However, significant differences between control and other gene combinations were only detected for these fatty acids’ levels in *Ot*DES6 + PSE + *Tc*DES5.

**Figure 6:**
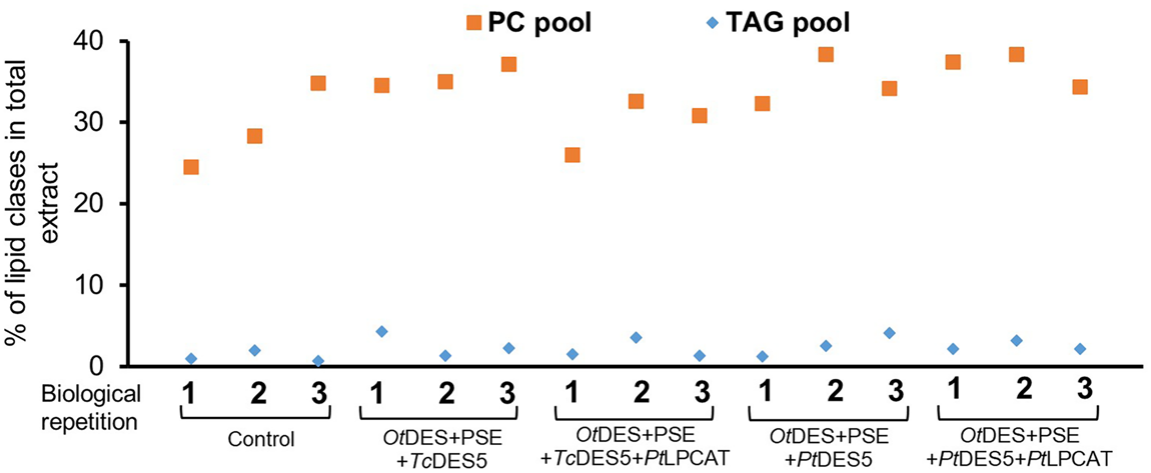
Distribution of phosphatidylcholine and triacylglycerol content in *Nicotiana benthamiana* leaves, caused by agroinfiltration of tested gene combinations. The graph sequentially represents results for control, *Ot*DES6 + PSE + *Tc*DES50, *Ot*DES6 + PSE + *Tc*DES5 + *Pt*LPCAT, *Ot*DES6 + PSE + *Pt*DES5 and *Ot*DES6 + PSE + *Pt*DES5 + *Pt*LPCAT. The dots indicate the content of the PC or TAG pools of each infiltrated sample, measured as the percentage of the whole extract separated on TLC plates. The mean value and standard deviation are presented in Table S6.

Our studied gene combinations did not only change the composition of fatty acids, but also changed the overall distribution of tested lipid pools. The PC pool content was increased from 29.2 to 35.6% for *Ot*DES6 + PSE + *Tc*DES50, to 29.8% for *Ot*DES6 + PSE + *Tc*DES5 + *Pt*LPCAT, to 35.0% for *Ot*DES6 + PSE + *Pt*DES5 and to 36.7% for *Ot*DES6 + PSE + *Pt*DES5 + *Pt*LPCAT. Whereas TAG pool increased on average 2-times compared to the control combination (Fig. 6, Table S5).

## Discussion

Until now many attempts have been made to produce transgenic plants enriched with VLC-PUFAs. They have focused on searching for the most promising and effective combinations of various ∆^6^-, ∆^5^-desaturases and ∆^6^-elongases, which were introduced into plants by both transient and stable expression methods^7,20–22^. The goal of our study was to verify next potential gene combinations aimed at elevated VLC-PUFAs biosynthesis, directed to the EPA production, and for the first-time verification of the *in planta* effect of simultaneous action of LPCAT enzyme native to marine microalga naturally producing EPA—*P. tricornutum.* In previous experiments we characterized this enzyme as a first enzyme with acyl-CoA:lysophosphatidylcholine acyltransferase activity from a diatom—*P. tricornutum*^12^. A fact of particular significance is that *Pt*LPCAT exhibited high substrate preference toward VLC-PUFAs, especially toward fatty acids from ω3-pathway. This finding suggests that *Pt*LPCAT can eliminate a metabolic bottleneck called “substrate dichotomy”, which has been postulated for many years, although clear evidence of its role in reverse reaction (transfer of FA from the *sn*-2 position of PC to acyl-CoA pool) is still required. “Substrate dichotomy” has been limiting efficient production of EPA and DHA in transgenic oilseed plants^1,8,9,11,21^. LPCAT enzymes exhibiting substrate specificity toward fatty acids from ω3-pathway were suspected to overcome this limitation, as they play an essential role in modifying acyl-CoA pool and phosphatidylcholine pool through forward and reverse reactions^23^. Previous studies verifying the role of another representative of acyl-CoA:lysophospholipid acyltransferases, a putative lysophosphatidic acid acyltransferase (LPAAT) from *Thraustochytrium sp* in the production of VLC-PUFAs in rapeseed led to the accumulation of EPA at the level of 15% of total fatty acids, however, the authors do not indicate that this is solely the effect of this acyltransferase^24^.

Prior to verifying *Pt*LPCAT action, we characterized the activity of three different ∆^6^-desaturases. Despite the previously confirmed activity of ∆^6^-desaturase from *P. tricornutum* in *Saccharomyces cerevisiae*^11,16^ and seeds of *Nicotiana tabacum* and *Linum usitatissimum*^8^*,* in our assays we did not observe its ability to produce GLA and SDA. Despite the use of Cauliflower mosaic virus 35S promoter or nos promoter no activity was noticeable. This result might be an effect of host and tissue specificity, previously described for seeds of different plants^8,20,25^. It may also be the result of the localization of this enzyme, which occurs natively to the ER or the fact that desaturases from microalgae can use not only the PC, but also PE and betaine lipids as substrates for desaturation^25^. Nevertheless, it does not preclude *Pt*DES6 for further application, and it was excluded from this study for the purpose of our experiment.

Two other tested ∆^6^-desaturases were active in *N*. *benthamiana* leaves. A lipid-linked desaturase from *R. stolonifera* was until now only characterized in the yeast *Pichia pastoris.* In native conditions it participates in production and accumulation of up to above 49% of GLA^15^. Our data shows that beside its ability to convert linoleic acid into GLA, it possesses the ability to produce SDA from linolenic acid. The second desaturase, which showed significantly elevated ability in the production of GLA and SDA was acyl-CoA-dependent desaturase from *O. tauri*. Its activity was previously noticed both in yeast and in plants^7,11,13,14^. The observed superior utilization of LA and ALA by *Ot*DES6 might be the result of its affinity toward the acyl-CoA, instead of lipid-bound acyls. Its more favorable in synthetic VLC-PUFAs biosynthesis in plants as the next step of this biosynthesis, the elongation, also utilizes these substrates and occurs in cytosol. This effect might be amplified by the efficient activity of endogenous LPCAT: supplying LA and ALA for the cytosol-based *Ot*DES6 desaturase and decreasing its availability for *Rs*DES6, which acts on membrane lipid-bound acyls. Exogenous introduction of *Pt*LPCAT further elucidates this effect. The production and accumulation of GLA and SDA was significantly elevated, especially for the *Ot*DES6 + *Pt*LPCAT TAG pool, where their content amounted to 13.1% and 19.1% of all fatty acids, respectively. GLA and SDA content increased by 1.8- and 1.5-times, respectively, in this combination, compared to the gene combination without *Pt*LPCAT co-expression. Simultaneously, reduced levels of ALA and LA in the PC pool were observed. It correlated with boosted GLA and SDA production and suggested that LPCAT transfer of acyl groups form PC to the acyl-CoA pool stimulates acyl-CoA desaturase action. The opposite situation was noted for *Rs*DES6 co-expressed with *Pt*LPCAT. While in none of the tested acyl-lipid pools, the content of GLA changed significantly, the content of SDA drastically decreased. In the PC pool it was not detectable, however still some amount, lower than for *Rs*DES6 action only, was present in the total lipid pool. It indicated that even though SDA synthesis via *Rs*DES6 occurred, probable efficient *Pt*LPCAT reverse action is also conducted. The observed differences in distribution of SDA in lipid pool can be the effect of different further ways of its utilization. Newly synthesized SDA in acyl-CoA, is probably directly incorporated into PC pool (if no elongation occurred), as a very efficient *Pt*LPCAT forward activity toward these fatty acids has been already confirmed^12^. From PC it is probably utilized for TAG biosynthesis via phospholipid:diacyglycerol biosynthesis (PDAT), as an elevated level of this SDA is observed in this pool. In case of SDA synthesized via phospholipid-linked desaturase action, it is probably first removed from the PC pool via reverse activity of LPCAT enzyme and it is later subjected to other acyltransferase action, or it can return to the PC pool, however this route reduced efficiency of SDA flux into TAG pool, as it can be incorporated into other lipids. This mechanism of SDA transfer is only a suggestion as tobacco acyltransferases substrate specificity toward these fatty acids was not tested. This finding indicates that, the co-action of acyl-lipid desaturase—*Ot*DES6 and *Pt*LPCAT can be potentially used for efficient production of oilseed plants producing GLA and SDA, as their significant levels were detected in the TAG pool, which is a major lipid pool in seeds, whereas tested lipid-linked desaturases do not act synergistically with LPCAT.

Based on the first part of this study we observed increased efficient production of the first two precursors of VLC-PUFAs, via *Pt*LPCAT co-expression with acyl-CoA desaturase. Therefore, for the plant incorporation of EPA biosynthesis pathway we used ∆^6^-desaturase from *O. tauri*, which we co-expressed with ∆^6^-elongases from *P. patens*. We designed two different gene combination in this way, each contained a distinct ∆^5^-desaturase: either from *Thraustochytrium sp.* or from *P. tricornutum.* Both applied combinations resulted in a successful production of EPA. In a previous study *Ot*DES6 + PSE + *Tc*DES5 was introduced into Arabidopsis through stable transformation, which allowed for the production of EPA amounting to 5.7% of all fatty acids presented in seeds^7^. Compared to this result our data obtained for TAG pool we obtained similar EPA production and higher proportion of all newly synthesized ω3 and ω6 fatty acids. Also comparing another tested combination: *Ot*DES6 + PSE + *Pt*DES5 with previous results derived from yeast the level of EPA production was very similar and amounted approximately to 4.3–4.7%, for total acyl-lipid in yeast and for fatty acids in the TAG pool of *N. benthamiana* leaves (Ref.^13^; presented data). Nevertheless, these analyses were conducted for distinct organisms, which needs to be taken into account, especially since yeast were exogenously supplemented in ALA, as they do not produce them naturally. As both used gene combinations gave positive results and were previously tested and confirmed in other organs or organisms, they were the best candidates for testing their co-expression with *Pt*LPCAT. The effect of *Pt*LPCAT overexpression was until now examined exclusively in *Yarrowia lipolytica*, where its co-expression with specific desaturases and elongases boosted EPA production^26^. In our study we also observed elevated accumulation of EPA, which was especially pronounced for the *Ot*DES6 + PSE + *Pt*DES5 + *Pt*LPCAT combination. In the total lipid content, EPA amount rose significantly from 0.8 to 1.4% (when *Pt*LPCAT was co-expressed). For *Ot*DES6 + PSE + *Tc*DES5 + *Pt*LPCAT, such increase was not observed, and the overall production of nonnative fatty acids declined. Nevertheless, the percentage of EPA in newly synthesized fatty acids pool still increased. The detected high level of EPA in TAG pool was equal to 2.9% for *Ot*DES6 + PSE + *Tc*DES5 + *Pt*LPCAT and 4.5% for *Ot*DES6 + PSE + *Pt*DES5 + *Pt*LPCAT (which accounted for 17% and 20.1% of newly synthesized fatty acids, respectively) is also an important basis for further research and stable transformation of oilseed plants. It should be also mentioned that the co-expression of *Pt*LPCAT also negatively affected accumulation of these fatty acids in TAG pool, what we previously observed for expression of phospholipid-linked desaturase from *R. stolonifer* and *Pt*LPCAT, as they might reduce availability of LC-PUFAs for further incorporation into TAG. However, the key aspect is that simultaneous co-expression of *Pt*LPCAT, with both tested gene combinations for EPA production, is always boosted the production and accumulation of ω3-LC-PUFAs. These increases were detected in total lipid content, for PC pools and for TAG pools. This increase fluctuated between 2 and 7% depending on the tested combination and lipid pool. It is a very significant finding as until now no efficient way to stimulate ω3- over ω6- pathways were detected, since so-far characterized desaturases are not specific just for none of these pathways. Nevertheless, expression of a desaturase utilizing as a substrate acyl-CoA can decreased accumulation of the omega-6 intermediates and partially circumvents the bottleneck of substrate availability^11,14^. This yields the possibility to boost desired ω3-VLC-PUFAs production in oilseeds. Nevertheless, it still does not fully solve the postulated bottleneck of “substrate dichotomy”. High accumulation of the intermediates persists. Their significant amount was also accumulated into the TAG pool. This may suggest that endogenous enzymes are highly specific for SDA and GLA, which was also noticed for gene combinations containing only p19, GFP and ∆^6^-desaturase from *O. tauri* in tobacco leaves. This result can be only host or organ specific, so further studies are needed. It is also important to look for other acyl-CoA:lysophospahtidycholine acyltransferases from organisms natively producing VLC-PUFAs to elucidate the *Pt*LPCAT role in ω3- and ω6-pathway. Another important issue which can be essential for determination of full potential of *Pt*LPCAT activity is its co-expression of additional native protein—acyl-CoA binding protein (ACBP), which role has been already determined as a protein facilitating the activity of acyltransferases^27^. The lack of this protein can be the reason for the partial, observed bottleneck. The ACBP from *P. triconutum* has yet to be characterized.

## Material and methods

### Plant material and growth conditions

*Nicotiana benthamiana* plants used for *Agrobacterium tumefaciens*-mediated infiltration were grown in a climate chamber. They were cultivated at 23 °C/20 °C for 13 h/11 h with the light (260 μmol/m^2^/s µmol photosynthetis photon flux density). Day/night conditions lasted for 16 h/8 h and relative humidity was 60%. After 4–5 weeks post-sowing and before flowering period, the middle leaves were used for agroinfiltration. After infiltration plants were grown under the same conditions mentioned above.

### Construction of plant expression vectors and *Agrobacterium tumefaciens* transformation

All tested genes were ordered as a synthetic codon optimized sequence (ThermoFisher Scientific) and were introduced by Golden Gate cloning method into the appropriate level 1 acceptor vector, which will allow for the further construction of functional EPA expression cassettes used for stable plant transformation, according to the method described by Engler et al.^28^. Constructed expression cassette were under the enhanced 35S, Cauliflower mosaic virus 35S promoter and OCS, octopine synthase terminator. The expectation was gene encoding p19 and GFP which were introduced by Gateway cloning in the pXZP393 vector. Genes, their definition, used abbreviations and database references are presented in Table 2.

**Table 2:** Description of genes used in the study.

Gene abbreviation	Enzymatic activity	Origin organism	Accession	Reference: activity confirmation
p19	RNA silencing suppressor	Artificial sequence	P69516.1	^29^
GFP	Reporter gene, green fluorescent protein	Tomato bushy stunt virus (strain ja6)	ABE28520	^30^
*Pt*DES6	∆^6^ lipid-linked desaturase	*Phaeodactylum tricornutum*	XM_002182865.1	^11,16^
*Rs*DES6	∆^6^ lipid-linked desaturase	*Rhizopus stolonifer* (strain YF6)	AY795076.1	^15^
*Ot*DES6	∆^6^ acyl-CoA desaturase	*Osterococcus tauri*	XM_003082530.1	^7,11,13,14^
*Pt*DES5	∆^5^ lipid-linked desaturase	*Phaeodactylum tricornutum*	GQ352540.1	^11,16^
*Tc*DES5	∆^5^ lipid-linked desaturase	*Thraustochytrium sp.* (ATCC21685)	AF489588	^18^
PSE	∆^6^ elongase	*Physcomitrium patens*	AF428243.1	^17^
*Pt*LPCAT	acyl-CoA:lysophosphatidylcholine acyltransferase	*Phaeodactylum tricornutum*	EEC48011.1	^12^

Constructed expression cassette with single tested gene were introduced into *A. tumefaciens* GV3101 by electroporation (2.4 kV, 25μF, 200Ω) using BioRad Gene Pulser Xcell electroporator. Electroporated cells were cultured in Luria–Bertani medium (LB) for 4 h at 28 °C with shaking. After incubation, cells were plate on agar plates containing ampicillin (100 μg/μl), rifampicin (50 μg/μl) and gentamycin (25 μg/μl) and grown for 2 days at 28 °C.

### Agroinfiltration of *Nicotiana benthamiana*

Agrobacterium colonies carrying tested gene constructs were inoculated in LB medium containing appropriate selection antibiotics and cultivated overnight at 28 °C at 200 rpm. Next day, acetosyringone was added to the final concentration equal to 100 µM and cultivation continued for additional 3 h. After this time, Agrobacterium cells were centrifuged 5 min at 3000×*g* and resuspended in 5 ml of infiltration buffer with pH 5.7 containing 5 mM MgCl_2_, 5 mM MES and 100 µM acetosyringone. In final infiltration media used for agroinfiltration OD_600_ of each culture, being part of given gene combination, was adjust to 0.2. The mixture of different Agrobacterium culture was infiltrated into tobacco 4–5 weeks old leaves using 1 ml plastic syringe. The transformed plants were placed back into the growth chamber. After 5 days, the infiltrated leaves areas were detected by GFP reporter fluorescence and excised. Collected material was frozen in liquid nitrogen and stored for further analysis at − 80 °C.

### Lipid extraction and analysis

Collected leaves material, prior to the lipid extraction, were freeze-dried for 3 days and weighted. The lipid extraction was conducted, according to the Blight and Dyer method^31^, by homogenization of freeze-dried leaves in 7 ml (2 × 3.75 ml) of chloroform:methanol (1:2; v:v) and 2.5 ml (2 × 1.25 ml) of 0.15 M acetic acid. The homogenates were transferred to glass tubes, filled with 2.5 ml of chloroform and 2.5 ml of distilled water, and centrifuged for 2 min at 1000×*g*. Bottom, chloroform fractions were collected, dried under nitrogen, and dissolved in 1 ml chloroform. The aliquots corresponding to 10 mg and 50 mg were used for fatty acids content assessment and for PC or TAG analysis, respectively.

The aliquots for fatty acid composition were dried under nitrogen and dissolved in methylation mixture to which 50 nmol of 17:0 was added as an internal standard. Prepared samples were subjected to methylation for 40 min at 90 °C. After methylation, formed fatty acids methyl esters were extracted by heptane and separated on CP-Wax 58 FFAP CB column (Agilent) in a gas chromatograph with a flame ionization detector (Agilent 8860 GC System).

For PC and TAG analysis, the aliquots were dried under nitrogen, dissolved in 50 µl of chloroform and applied to TLC plates (silica gel 60, Merck). Completely dried plates were placed in the TLC chamber filled with appropriate mobile phase. For TAG separation phase containing heptane, diethyl ether and acetic acid (70:30:1; v:v:v) was used, while for PC separation phase composed of chloroform, methanol, acetic acid and distilled water (90:15:10:2.5; v:v:v:v). After separation, when plates were well dried, they were sprayed with primuline and visualized under UV light to detect separated lipid classes. Silica gel corresponding to phosphatidylcholine and triacylglycerol were scraped off and subjected to methylation. Further steps were conducted as described above for analysis of total fatty acid composition.

### Ethic approval and statement according to used methods

All experiments were performed in accordance with relevant institutional and national guidelines and regulations.

### Statement related to used plant material

Formal identification of the plant material used in your study was done by Sylwia Klińska-Bąchor and Kamil Demski. Research using *Nicotiana benthamiana* was carried out at Swedish University of Agricultural Science in Alnarp, which has permission to cultivate and conduct research using this plant.

### Supplementary Information

Supplementary Tables.

## Data Availability

The datasets used and/or analyzed during the current study are available from the corresponding author on reasonable request.
