# Supplementary material for: Metabolic engineering of omega-3 long chain polyunsaturated fatty acids in plants using different ∆6- and ∆5-desaturases co-expressed with LPCAT from the marine diatom Phaeodactylum tricornutum
Source: Sci Rep. 2024 Apr 25;14:9512. doi: 10.1038/s41598-024-60141-3 (PMC11045822; doi:10.1038/s41598-024-60141-3)
Supplement: Supplementary file 1 — Supplementary Tables. [file 41598_2024_60141_MOESM1_ESM.pdf]

Supplementary Materials

Metabolic engineering of omega-3 long chain polyunsaturated fatty acids in plants using different ∆6- and ∆5-desaturases co-expressed with LPCAT from the marine diatom *Phaeodactylum tricornutum*

Sylwia Klińska-Bąchor[1,2,*], Kamil Demski[2], Yangmin Gong[3], Antoni Banaś[1]

[1] Intercollegiate Faculty of Biotechnology, University of Gdańsk and Medical University of Gdańsk, 80-307 Gdańsk, Poland

[2] Department of Plant Breeding, Swedish University of Agricultural Sciences, Box 190, 23422, Lomma, Sweden

[3] Oil Crops Research Institute of Chinese Academy of Agricultural Sciences, 430062 Wuhan, China

[*]Correspondence author: email: sylwia.klinska@ug.edu.pl; address: Intercollegiate Faculty of Biotechnology, University of Gdańsk and Medical University of Gdańsk, ul Abrahama 58, 80-307 Gdańsk, Poland

Table S1 Fatty acid composition in acyl-lipid, phosphatidylcholine and triacylglycerol pools of *Nicotiana benthamiana* leaves derived through agroinfiltration with gene combinations encoding *Pt*LPCAT and *Pt*DES6. Mean values and standard deviations of three independent biological replicates are presented. Asterisks denote statistical significance compared to control calculated in two-tailed Student's t-test: * – p≤0.05; ** - p≤0.01; *** - p≤0.001.

	FA in total extract			FA in PC pool		FA In TAG pool	
	**Control**	***Pt*LPCAT**	***Pt*DES6**	**Control**	***Pt*LPCAT**	**Control**	***Pt*LPCAT**
**16:0**	14.5±0.2	15.9±0.7	14.9±0.3	21.8±0.9	21.6±1.3	34.8±2.1	29.2±2.7
**16:1$^{\Delta7+\Delta9}$**	4.0±0.1	3.1±0.2***	3.6±0.1	-	-	-	-
**16:3$^{\Delta7,10,13}$**	7.1±0.1	6.2±1.0*	7.0±0.2	-	-	-	-
**18:0**	2.1±0.03	2.4±0.4*	2.3±0.1	3.3±0.1	3.6±0.3	5.1±1.9	5.6±0.3
**18:1$^{\Delta9}$**	1.2±0.1	1.1±0.8	1.1±0.3	1.7±0.2	1.4±0.7	1.9±0.2	2.0±0.6
**18:1$^{\Delta11}$**	0.7±0.02	0.8±0.1	0.6±0.4	0.9±0.03	1.0±0.1	-	-
**18:2$^{\Delta9,12}$**	11.3±0.1	11.7±3.1	11.2±0.8	12.7±0.6	12.6±1.2	20.3±0.02	19.4±1.9
**18:3$^{\Delta9,12,15}$**	58.6±0.2	58.3±4.8	58.6±0.9	59.6±0.9	59.8±1.5	35.8±1.9	42.8±1.1**
**20:0**	0.5±0.02	0.6±0.1	0.6±0.03	-	-	2.0±0.8	1.0±0.2

Table S2 Mean value and standard deviation of phosphatidylcholine and triacylglycerol content in *Nicotiana benthamiana* leaves, caused by the action of three different exogenous gene combinations: *Pt*LPCAT, *Ot*DES6 and *Ot*DES6 with *Pt*LPCAT. Single biological replicates presented in Figure 4. Asterisks denote statistical significance compared to control calculated in two-tailed Student's t-test: * – p≤0.05; ** - p≤0.01.

**Gene combinations**	**Control**	***Pt*LPCAT**	***Ot*DES6**	***Ot*DES6 +*Pt*LPCAT**
**PC**	27.3 ±2.5	33.2* ±1.4	35.7* ±2.2	34.2* ±0.4
**TAG**	1.2 ±0.3	3.4 ±2.0	7.4** ±1.2	5.6** ±1.4

Table S3 Fatty acid composition of lipid pools in *Nicotiana benthamiana* leaves remained after phosphatidylcholine and triacylglycerol separation, derived through agroinfiltration with gene combinations aimed at producing GLA (18:3$\Delta^{6,9,12}$) and SDA (18:4$\Delta^{6,9,12,15}$). Mean values and standard deviations of three independent biological replicates are presented. Asterisks denote statistical significance compared to control calculated and letter a indicate statistical difference between combination with desaturase and desaturase with *Pt*LPCAT calculated in two-tailed Student's t-test: * – p≤0.05; ** - p≤0.01; *** - p≤0.001; a - p≤0.05.

	mol% of FA in remaining extract after TAG pool separation					mol% of FA in remaining extract after PC pool separation				
	**Control**	***Rs*DES6**	***Rs*DES6 +*Pt*LPCAT**	***Ot*DES6**	***Ot*DES6 +*Pt*LPCAT**	**Control**	***Rs*DES6**	***Rs*DES6 +*Pt*LPCAT**	***Ot*DES6**	***Ot*DES6+ *Pt*LPCAT**
**16:0**	14.6±0.4	15.3±0.9	15.0±0.7	17.8±0.8**	15.7±0.9	8.9±0.3	14.8±1.3**	16.2±1.4***	17.3±1.1***	14.8±1.2**
**16:1$^{\Delta7+\Delta9}$**	8.8±0.7	7.5±0.6	8.1±0.1	6.4±0.5*	7.4±0.4	8.8±0.1	11.5±1.3	9.7±0.5	9.5±0.7	12.2±0.9
**16:3$^{\Delta7,10,13}$**	7.1±0.2	6.2±0.1*	6.4±0.1*	5.4±0.3**	7.1±0.8	11.9±0.5	7.6±0.01**	7.0±0.2**	6.8±0.5***	8.0±0.9**
**18:0**	2.0±0.1	2.2±0.1	2.2±0.04*	2.7±0.2**	2.2±0.2$_{a}$	0.9±0.05	1.9±0.2**	2.5±0.1**	2.3±0.2***	1.8±0.1***
**18:1$^{\Delta9}$**	1.0±0.1	1.4±0.1	1.9±0.3*	1.3±0.2	1.0±0.1	0.6±0.04	1.2±0.2*	2.2±0.4	1.3±0.2*	1.0±0.1*
**18:1$^{\Delta11}$**	0.7±0.01	0.8±0.02	0.7±0.02*	0.9±0.04**	0.7±0.1	0.4±0.01	0.7±0.1	0.7±0.01**	0.7±0.03***	0.7±0.1**
**18:2$^{\Delta9,12}$**	9.5±0.3	10.4±1.3	12.8±1.3*	9.7±0.4	7.9±0.7$^{*}_{a}$	7.9±0.4	9.9±1.4	14.6±1.9	9.1±0.6	7.3±0.9
**18:3$^{\Delta6,9,12}$**	-	0.4±0.1	0.3±01	2.1±0.9	3.1±1.8	-	0.4±0.1	0.4±0.2	2.3±1.1	3.0±1.7
**18:3$^{\Delta9,12,15}$**	56.0±0.6	55.2±2.1	51.7±2.1	50.0±2.5*	49.8±2.6*	59.8±0.6	51.4±2.1*	45.7±3.5	46.0±3.0**	46.2±2.3***
**18:4$^{\Delta6,9,12,15}$**	-	0.4±0.1	0.5±0.1	3.3±1.0	4.8±1.5	-	0.3±0.03	0.2±0.1	4.2±1.4	4.7±1.2
**20:0**	0.3±0.03	0.3±0.04	0.3±0.04	0.3±0.03	0.3±0.01	0.6±0.2	0.3±0.03	0.7±0.1	0.4±0.03	0.3±0.02$_{a}$

Table S4 Fatty acid composition of lipid pools in *Nicotiana benthamiana* leaves remained after phosphatidylcholine and triacylglycerol separation, derived through agroinfiltration with gene combinations aimed at producing EPA (20:5$^{\Delta5,8,11,14,17}$). Mean values and standard deviations of three independent biological replicates are presented. Asterisks denote statistical significance compared to control calculated and letter a indicate statistical difference between combination without *Pt*LPCAT and with *Pt*LPCAT, calculated in two-tailed Student's t-test: * – p≤0.05; ** - p≤0.01; *** - p≤0.001; a - p≤0.05.

	**mol% of FA in remaining extract after TAG pool separation**					**mol% of FA in remaining extract after PC pool separation**				
	**Control**	***Ot*D6+PSE+ *Tc*D5**	***Ot*D6+PSE+ *Tc*D5 +*Pt*LPCAT**	***Ot*D6+PSE+ *Pt*D5**	***Ot*D6+PSE+ *Pt*D5 +*Pt*LPCAT**	**Control**	***Ot*D6+PSE+ *Tc*D5**	***Ot*D6+PSE+ *Tc*D5+*Pt*LP CAT**	***Ot*D6+PSE+ *Pt*D5**	***Ot*D6+PSE+ *Pt*D5 +*Pt*LPCAT**
**16:0**	12.9±0.8	14.7±0.5*	15.8±0.3$^{**}_{a}$	15.0±0.1*	15.6±0.3$^{**}_{a}$	12.0±0.6	14.3±1.3	13.9±0.8*	12.0±1.9	13.1±0.5*
**16:1$^{\Delta7+\Delta9}$**	7.0±1.2	7.1±1.5	7.7±0.3	7.5±0.1	7.7±0.2	10.7±1.9	9.3±2.8	10.4±0.5	9.5±1.3	10.2±0.4
**16:3$^{\Delta7,10,13}$**	8.5±0.9	6.5±1.2	7.1±0.2	6.9±0.2	6.8±0.5	9.4±1.3	8.5±1.1	8.5±0.3	8.0±1.0	8.6±0.9
**18:0**	1.4±0.2	2.2±0.2*	2.1±0.1*	2.0±0.1*	2.1±0.1*	1.6±0.3	1.8±0.2	1.7±0.1	1.6±0.2	1.7±0.1
**18:1$^{\Delta9}$**	0.8±0.02	1.0±0.2	0.8±0.1	1.1±0.02***	1.0±0.1*	0.8±0.1	0.8±0.2	0.8±0.1	0.9±0.1	0.8±0.1$_{a}$
**18:1$^{\Delta11}$**	0.6±0.07	0.9±0.1	0.9±0.03	0.8±0.04***	0.9±0.01*	0.6±0.1	0.8±0.2	0.8±0.1	0.7±0.1	0.8±0.1$_{a}$
**18:2$^{\Delta9,12}$**	9.7±0.04	8.8±1.4	8.4±0.5	8.7±0.2***	8.9±0.6	10.0±1.6	7.7±1.6	7.8±0.3	7.1±1.2	7.8±0.6
**18:3$^{\Delta6,9,12}$**	-	1.5±0.8	1.1±0.2	1.6±0.5	1.6±0.7	-	1.4±0.8	1.1±0.3	1.6±0.7	1.6±0.5
**18:3$^{\Delta9,12,15}$**	59.0±1.3	54.9±2.8**	54.3±0.3*	52.0±1.9**	52.6±1.2**	54.6±0.1	51.5±1.8	51.4±1.6	44.97±6.4	50.5±1.2*
**18:4$^{\Delta6,9,12,15}$**	-	0.5±0.1	0.4±0.04	2.2±0.6	0.4±0.1$_{a}$	-	2.0±0.9	1.9±0.6	2.1±0.9	2.5±0.6
**20:0**	0.2±0.1	0.3±0.01	0.3±0.02	0.3±0.03	0.3±0.02	0.3±0.04	0.3±0.03	0.3±0.01	0.3±0.07	0.3±0.03
**20:3$^{\Delta8,11,14}$**	-	0.6±0.4	0.3±0.1	0.8±0.3	0.7±0.4	-	0.6±0.4	0.4±0.1	0.7±0.4	0.7±0.4
**20:4$^{\Delta5,8,11,14}$**	-	0.1±0.01	0.1±0.01	0.1±0.01	0.1±0.01	-	0.1±0.01	0.05±0.01	-	0.1±0.02
**20:4$^{\Delta8,11,14,17}$**	-	0.2±0.1	0.2±0.1	0.2±0.1	0.3±0.1	-	0.2±0.1	0.2±0.1	0.3±0.2	0.3±0.1
**20:5$\Delta^{5,8,11,14,17}$**	-	0.9±0.3	0.5±0.2	0.9±0.3	1.0±0.4	-	0.8±0.5	0.6±0.1	0.9±0.5	1.0±0.2

Table S5 Mean value and standard deviation of phosphatidylcholine and triacylglycerol content in *Nicotiana benthamiana* leaves, caused by agroinfiltration of tested gene combinations. The graph sequentially represents results for control, *Ot*DES6+PSE+*Tc*DES50, *Ot*DES6+PSE+*Tc*DES5 +*Pt*LPCAT, *Ot*DES6+PSE+*Pt*DES5 and *Ot*DES6+PSE+*Pt*DES5 +*Pt*LPCAT. Single biological replicates presented in Figure 6.

**Gene combinations**	**Control**	***Ot*D6+PSE +*Tc*D5**	***Ot*D6+PSE +*Tc*D5 +*Pt*LPCAT**	***Ot*D6+PSE +*Pt*D5**	***Ot*D6+PSE +*Pt*D5 +*Pt*LPCAT**
**PC**	29.2 ±4.3	35.6 ±1.2	29.8 ±2.8	35.0 ±2.5	36.7 ±1.7
**TAG**	1.2 ±0.6	2.6 ±1.3	2.1 ±1.0	2.6 ±1.2	2.5 ±0.5

Table S6 Standard deviations of fatty acid composition in acyl-lipid of *Nicotiana benthamiana* leaves obtained through agroinfiltration with gene combinations aimed at producing EPA (20:5$^{\Delta 5,8,11,14,17}$). Mean values presented in Figure 5.

	SD of mol% FA in total extract														
	**16:0**	**16:1$^{\Delta 7+\Delta 9}$**	**16:3$^{\Delta 7,10,13}$**	**18:0**	**18:1$^{\Delta 9}$**	**18:1$^{\Delta 11}$**	**18:2$^{\Delta 9,12}$**	**18:3$^{\Delta 6,9,12}$**	**18:3$^{\Delta 9,12,15}$**	**18:4$^{\Delta 6,9,12,15}$**	**20:0**	**20:3$^{\Delta 8,11,14}$**	**20:4$^{\Delta 5,8,11,14}$**	**20:4$^{\Delta 8,11,14,17}$**	**20:5$^{\Delta 5,8,11,14,17}$**
**Control**	0.9	0.4	0.4	0.1	0.1	0.1	0.5		0.7		0.01				
***Ot*D6+PSE +*Tc*D5**	1.4	0.7	0.7	0.3	0.2	0.1	1.9	0.3	0.9	0.3	0.01	0.4	0.02	0.1	0.2
***Ot*D6+PSE +*Tc*D5 +*Pt*LPCAT**	0.7	0.2	0.3	0.1	0.1	0.03	0.6	0.2	0.7	0.3	0.03	0.1	0.01	0.1	0.1
***Ot*D6+PSE +*Pt*D5**	0.4	0.4	0.2	01	0.01	0.04	0.2	0.5	1.8	0.6	0.06	0.5	0.03	0.2	0.1
***Ot*D6+PSE +*Pt*D5 +*Pt*LPCAT**	0.3	0.5	0.7	0.1	0.1	0.1	0.8	0.1	1.2	0.2	0.06	0.4	-	0.2	0.2
